# Novel Method for Safeguarding Personal Health Record in Cloud Connection Using Deep Learning Models

**DOI:** 10.1155/2022/3564436

**Published:** 2022-03-19

**Authors:** Sarvesh Kumar, Mohammed Abdul Wajeed, Rajashekhar Kunabeva, Nripendra Dwivedi, Prateek Singhal, Sajjad Shaukat Jamal, Reynah Akwafo

**Affiliations:** ^1^Department of Computer Science and Engineering, BBD University, Lucknow, India; ^2^Department of Computer Science and Engineering, Mahatma Gandhi Institute of Technology, Gandipet, Hyderabad, India; ^3^Department of Electronics and Communication Engineering, GM Institute of Technology, Visveswaraya Technological University, Karnataka, India; ^4^Department of Computer Science/IT, Institute of Management Studies, Ghaziabad, Uttar Pradesh, India; ^5^Department of Computer Science and Engineering, Sagar Institute of Research & Technology-Excellence, Bhopal, Madhya Pradesh, India; ^6^Department of Mathematics, College of Science, King Khalid University, Abha, Saudi Arabia; ^7^Bolgatanga Technical University, Department: Electrical and Electronics Engineering, Sumbrungu, Ghana

## Abstract

It is a new online service paradigm that allows consumers to exchange their health data. Health information management software allows individuals to control and share their health data with other users and healthcare experts. Patient health records (PHR) may be intelligently examined to predict patient criticality in healthcare systems. Unauthorized access, privacy, security, key management, and increased keyword query search time all occur when personal health records (PHR) are moved to a third-party semitrusted server. This paper presents security measures for cloud-based personal health records (PHR). The cost of keeping health records on a hospital server grows. This is particularly true in healthcare. As a consequence, keeping PHRs in the cloud helps healthcare institutions save money on infrastructure. The proposed security solutions include an optimized rule-based fuzzy inference system (ORFIS) to determine the patient's criticality. Patients are classified into three groups (sometimes known as protective rings) based on their severity: very critical, less critical, and normal. In trials using the UCI machine learning archive, the new ORFIS outperformed existing fuzzy inference approaches in detecting the criticality of PHR. Using a graph-based access policy and anonymous authentication with a NoSQL database in a private cloud environment improves data storage and retrieval efficiency, granularity of data access, and response time.

## 1. Introduction 

**Figure 1:**
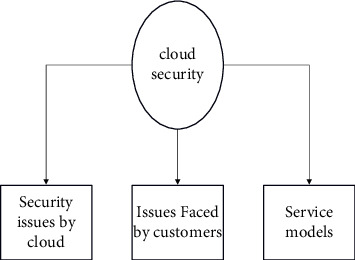
Cloud security issues.

Cloud computing is a fast-developing technology that has sparked a slew of technical advancements throughout the information technology sector. It has brought forth unprecedented advancement in a variety of fields. However, as a result of this remarkable expansion, it is susceptible to a wide range of security vulnerabilities, which may harm both the suppliers and the customers of cloud-based services. Cloud-based security concerns largely revolve around the protection of information travelling through and being kept in the cloud, as well as the availability of information, access to information, and privacy of information. A number of different ways to data encryption and service authentication have been developed in order to address these concerns. This section investigates and discusses a variety of such security challenges, as well as potential security solutions that have been used in the industry to enhance cloud security [1]. The taxonomy of cloud security is shown in Figure 1. Cloud storage solutions are confronted with several security concerns in cloud service models such as infrastructure as a service (IaaS), platform as a service (PaaS), software as a service (SaaS), and deployment patterns such as private, public, and hybrid clouds, among other scenarios [2]. These challenges are divided into two categories: security issues encountered by cloud service providers (CSP) and security issues faced by cloud service customers (CSC). The cloud provider must focus on the infrastructure and data security of its customers, while the user must guarantee that their application is strengthened by using secure authentication methods.

Figure 1 represents cloud security. If the cloud service provider wants to prevent unwanted on-site access to CSC data, the actual location of the cloud data centre must be protected by security measures. It is impossible to safeguard data against physical theft with firewalls and encryption alone. Because the CSP is in charge of the physical infrastructure, they should establish and maintain proper infrastructure controls, such as staff training, physical location security, and network firewalls, as well as monitor and maintain these controls. Another point to emphasize is that the CSP is not only responsible for storing and processing data in certain countries but also for complying with the privacy requirements of those nations [3]. The failures linked with the hardware, technology, and services supplied by the CSP are considered to be these risks. Resource sharing, isolation issues, and dangers associated with switching cloud service providers, or portability, are all concerns in the public cloud because of its multi-tenancy capabilities. It is recommended that CSP do routine maintenance and audits on the infrastructure. Inadequate understanding of the applicable jurisdiction, changes in the applicable jurisdiction, contract provisions that are illegal, and continuous legal disputes are all examples of risks related to the law. For example, depending on where you live, certain CSP may be required by law to send over sensitive information if the government so requests it from them [4]. The hazards associated with data security are many and must be taken into account. Data integrity, confidentiality, and availability are the three most important attributes that must be maintained at all times.

Existing research in the literature on personal health records systems has concentrated on decision support systems, security, privacy preservation, anonymization, and access control, among other things. The use of cloud-based online service models to access health information in a safe way has recently been popular among academics [5]. Such cloud-based solutions raise concerns about security and privacy, which have so far remained unresolved. There is currently no reliable system for processing healthcare records in the cloud that incorporates criticality analysis, secures access using attribute-based encryption techniques, and thereby addresses the key management difficulties that exist in the cloud environment. In addition to compromising privacy, the existing approach is unable to withstand collusion and replay assaults. In order to address these concerns, this study presents security techniques for accessing personal health records (PHR) in the cloud [6]. Patient health records (PHR) are first established as a basic storage service but are eventually upgraded to a cloud-based healthcare system that can be controlled by patients themselves.

To a large part, the present PHR system is focused on facilitating quick access to health information, illness management, and information exchange. Some of the most important parts of big data processing have been introduced from system and application perspectives, and these approaches include cloud computing platforms, cloud architectures, and databases as well as data storage schemes [7]. Instead of using the knowledge-rich content that is concealed inside the database to make clinical decisions, physicians frequently rely on their perspective and experience rather than on their perception and experience. Medical diagnosis is a critical but difficult activity that must be completed precisely and quickly; thus, the automation of this process would be quite beneficial. As a result, it is necessary to give an efficient and intelligent criticality analysis in order to identify the state of the patients in order to provide aid during an emergency [8].

Furthermore, the PHR is outsourced to a third-party server that is only partially trustworthy [9]. As a result, there are security and privacy concerns about the patient's sensitive health information. A cloud-based health information system has been the subject of current research in the healthcare arena, which has raised concerns about privacy and security problems [10]. The safe exchange of health information among individuals in a cloud setting continues to be a difficult problem to solve. This study focuses on security schemes and privacy preservation techniques to allow the safe exchange of personal health information in the cloud, in order to solve this difficulty in the cloud environment [11]. The term “access structure” is used often in the context of the healthcare delivery system. Patient health information may be shared between medical specialists, hospital organizations, academics, and health policymakers using the cloud as a platform for exchanging information [12]. Because of this, it is necessary to implement an access control mechanism for PHR data that is appropriate for the situation. In the current attribute-based encryption system (ABE) [2], the records are encrypted with users' private key attributes and access policy. which is stored in the cloud.

The decryption of health data can be done only when the users have an appropriate matching set of attributes. Anonymous authentication [3] is a phenomenon in which user authentication is done without the knowledge of users' identity, before storing the PHR in the cloud. This is achieved in the proposed leveraged decentralized multiadministrator attribute-based signature (L-D-MA-ABS). However, such cloud-based multiauthority attribute-based encryption schemes are vulnerable to attacks. Instead of using the knowledge-rich content that is concealed inside the database to make clinical decisions, physicians frequently rely on their perspective and experience rather than on their perception and experience. A collusion attack throws light on a specific type of attack in which no two users can collude on the access of data or perform self-authentication if they are individually not authorized [5].

Because of this, it is necessary to solve current challenges such as access control, authentication, and privacy protection at once. In order to address these concerns, this study offers an enhanced decentralized anonymous authentication with graph-based access structure that is decentralized but not anonymous (ED-AA-GAS). The proposed ED-AA-GAS is based on a unique method known as leveraged decentralized multiauthority attribute-based signature (L-D-MA-ABS), which allows only legitimate users to decode the stored health information in the cloud using the appropriate credentials and credentials. Furthermore, it makes it easier to do CRUD (creation, reading, updating, and deletion) actions on data that is stored in the cloud. The approach of anonymous authentication using leveraged decentralized multiauthority attribute-based signature (L-D-MA-ABS) is included in the proposed EAAGAS in order to overcome the current issues raised in the previous study. It is a challenging issue to efficiently communicate and search for outsourced health data in a safe environment. Searchable attribute-based encryption (SABE) makes it possible to search for keywords in the encrypted text, which saves time in the processing of keyword queries, which saves time in the search process. This study presents an improved SABE (ISABE) for PHR in the cloud in order to reduce the amount of time spent searching for and retrieving linked health characteristics from PHR databases. Improved ABE algorithms are being developed as part of this study to protect the privacy, trustworthiness, and security of users in a multiowner context. In this paper, we offer a graph-based access structure with anonymous authentication, which provides an efficient fine-grained access control mechanism, as well as an enhanced Searchable ABE, which is designed to reduce the search time of PHR.

The vital parameters of patients are subjected to fuzzy logic analysis in order to assess their criticality degree in the PHR system. An enhanced fuzzy inference system (FIS) known as an optimized rule-based fuzzy inference system (ORFIS) is presented to improve the FIS for effective rule search in the fuzzy rule base. The ORFIS is an acronym for optimized rule-based fuzzy inference system. The vital patient records are secured using enhanced attribute-based encryption methods, which protect the confidentiality of the information. The encrypted PHR is sent to the cloud, which raises concerns about security, privacy, and access management.

Electronic health records (EHRs) offer several advantages. Furthermore, EHRs can minimize medical mistakes and adverse events, increase patient security, and promote health system planning, reform, and research. Patients who have access to EHRs may easily renew prescriptions and schedule appointments. The following is a list of projected EHR benefits.

Patient care should be supported and improved at right time by increasing professional productivity while lowering administrative costs associated with healthcare delivery and financial administration. Consider future improvements in healthcare technology such as health-care policy, administration, and funding can lead good healthcare system. Access to patient records is secured and confidential in the proposed healthcare system.

Charts that have been misplaced in the organisation take special care to maintain consistency. Paperwork, documentation errors, and filling activities have all been reduced.

Coding efficiency is important considerations to secure the online patient records. Prescription mistakes, medicine interactions, and patient allergies are intimated immediately in the form of alerts.

To be functional, the EHR system must benefit all cardholders engaged. Among the projected advantages are improved confidentiality and security of all health-related data. An EHR is critical to a unified healthcare delivery system. To sustain this system, cardholders must benefit.

## 2. Background Study

Because of this, it is necessary to solve current challenges such as access control, authentication, and privacy protection at once. In order to address these concerns, this study offers an enhanced decentralized anonymous authentication with graph-based access structure that is decentralized but not anonymous (ED-AA-GAS). The proposed ED-AA-GAS is based on a unique method known as leveraged decentralized multiauthority attribute-based signature (L-D-MA-ABS), which allows only legitimate users to decode the stored health information in the cloud using the appropriate credentials and credentials. Furthermore, it makes it easier to do CRUD (creation, reading, updating, and deletion) actions on data that is stored in the cloud. The approach of anonymous authentication using leveraged decentralized multiauthority attribute-based signature (L-D-MA-ABS) is included in the proposed ED-AA-GAS in order to overcome the current issues raised in the previous study. It is a challenging issue to efficiently communicate and search for outsourced health data in a safe environment. Searchable attribute-based encryption (SABE) makes it possible to search for keywords in the encrypted text, which saves time in the processing of keyword queries, which saves time in the search process. This work offers an improved SABE (ISABE) for personal health records (PHR) in the cloud in order to minimize the amount of time spent looking for and retrieving connected health features from personal health records (PHR). Improved ABE algorithms are being developed as part of this study to protect the privacy, trustworthiness, and security of users in a multiowner context. In this paper, we offer a graph-based access structure with anonymous authentication, which provides an efficient fine-grained access control mechanism, as well as an enhanced Searchable ABE, which is designed to reduce the search time of PHR.

The vital parameters of patients are subjected to fuzzy logic analysis in order to assess their criticality degree in the PHR system. An enhanced fuzzy inference system (FIS) known as an optimized rule-based fuzzy inference system (ORFIS) is presented to improve the FIS for effective rule search in the fuzzy rule base. The ORFIS is an acronym for optimized rule-based fuzzy inference system. The vital patient records are secured using enhanced attribute-based encryption methods, which protect the confidentiality of the information. The encrypted PHR is sent to the cloud, which raises concerns about security, privacy, and access management represented in Table 1.

Using the revocable attribute-based ring signature scheme with constant size signature [12] message signing on behalf of a ring of prospective signers is accomplished via the use of a ring signature scheme. Meanwhile, attribute-based signatures, in which each user is specified by a collection of characteristics, are also used to provide anonymous authentication in order to protect sensitive information. In this paper, we describe an attribute-based ring signature technique with a constant size signature that uses attribute-based technology and is based on the ring signature. The method is revocable and has a continuous size signature [13]. The method is revocable and has a continuous size signature. In this method, the data owner signs messages with high levels of anonymity, and the attribute authority has the ability to remove the anonymity of a signature when it is required. As a result, the genuine signer may still build a ring at his discretion, but he must be held accountable for his signature. This system is also capable of providing collusion resistance [14]. Furthermore, the scheme's implementation is efficient since it is based on attributes rather than certificate-based authentication. However, the size of the signature, as well as the computing cost of the signing and verifying process, remains constant and is only dependent on the number of ring members in the system [15]. In light of the extensive study, source authentication is one of the concerns that should be examined in the field of information security. In order to ensure the authenticity of information sources on the Internet, a variety of sophisticated signature techniques, including the ABS, have been made available to users [16]. However, since the establishment of a ring among users is very difficult, the method can only enable the signature of a limited number of characteristics. The attribute-based signature technique is an urgent cryptographic fundamental that provides customers with a more intense approach to controlling their security by using attributes [17]. The attribute-based multisignature scheme (ABMS) is being suggested because of the way it may reduce the amount of data transfer required to send attribute-based signatures and the amount of time required to confirm the sign in the meanwhile. In attribute-based multisignature scheme in the standard model, they described a plan known as characteristic-based multi-signature plan (ABMS), formalized the model of ABMS, and provided a security model for ABMS [18]. They also formalized the model of ABMS and provided a security model for ABMS.

Although classic attribute-based encryption (ABE) technology ensures the secrecy of the data [13], it has a number of limitations when it comes to searching for information. The notion of searchable encryption (SE) was developed in order to make data searching in the ciphertext more convenient [14]. It is a cryptographic basic approach that maintains the security of outsourced data by encrypting it while also allowing for keyword search on the ciphertexts to be performed on them. The searchable attribute-based mechanism [15] allows for searching on the encrypted ciphertext while also allowing for the updating of the associated search keyword(s) even after the material has been distributed.

Existing research in the literature on personal health records systems has concentrated on decision support systems, security, privacy preservation, anonymization, and access control, among other things. The use of cloud-based online service models to access health information in a safe way has recently been popular among academics [19]. Such cloud-based solutions raise concerns about security and privacy, which have so far remained unresolved. There is currently no reliable system for processing healthcare records in the cloud that incorporates criticality analysis, secure access using attribute-based encryption techniques, and thereby addresses the key management difficulties that exist in the cloud environment [9]. In addition to compromising privacy, the existing approach is unable to withstand collusion and replay assaults. In order to address these concerns, this study presents security techniques for accessing personal health records (PHR) in the cloud. PHR is first established as a basic storage service, but it is eventually changed to a cloud-based healthcare system that patients may administer themselves [20]. To a large part, the present PHR system is focused on facilitating quick access to health information, illness management, and information exchange.

Despite these advantages, EHRs are not yet mainstream in today's healthcare systems. EHR technology is still a hot issue for debate in healthcare technology and management publications, despite the IOM's advice to use EHRs being over a decade-old cost, leadership, suppliers keeping up with customers' expectations, and deficiencies in public policy, standards, security, and a proper definition have hindered healthcare executives from implementing EHR technology [1, 2]. Following are the primary stages to consider while moving from a manual medical record system to an electronic health record.

1. They need appropriate technical knowledge to choose an EHR system. Health information management, health informatics, and electronic records should be strong areas of expertise.
2. Not having an IT background is not enough. They must comprehend both the manual and electronic health record environments.
3. They should be familiar with systems in underdeveloped nations.
4. Some nations have restrictions that may hinder EHR deployments, such as lack of funding, technical assistance, electrical supply, and skilled employees.

Software, hardware, infrastructure development and maintenance, implementation, education, planning, and administration are all funded. Purchase of workstations is a hardware expense, as is the creation or purchase of software. Services such as help desk operations and network maintenance are included in infrastructure development expenses [3, 4]. Implementation expenses include training, overtime involved with inputting patient data, company interruption during the transition, employee reluctance to change, and lost productivity. Inadequate medical record documentation has long been an issue in many institutions/countries. Incomplete, inadequate, or bad documentation and nonstandard terminology are issues.

No migration to an electronic health record will be effective unless documentation issues are addressed and healthcare providers are trained. Input data should be accurate, and alert message should be sent immediately during emergency.

## 3. Proposed Methodology

Many PHR strategies to assess illness severity fail in decision-making [16]. Thus, an intelligent decision-making system is required to classify PHRs according to patient criticality. ORFIS, an optimized rule-based fuzzy inference system, is presented to assess patient severity. Patients are divided into three categories: very critical, less critical, and normal. Finally, the encrypted data is cloud-based. When PHRs are moved to the cloud, several security issues occur. Thus, accessing sensitive patient health information must be secured [5, 6].

**Figure 2:**
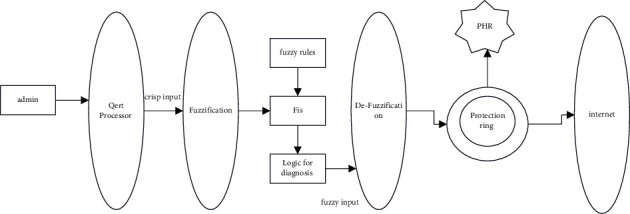
Proposed architecture for criticality analysis and secure access.

The PHR setup is broken into two phases: admin and user. Admins may store PHR on the cloud. These PHRs are given into the fuzzy expert system to assess patient severity. Once the patient's criticality is recognized, the PHR may be kept in the cloud protection ring. It allows the doctor to access PHR. Doctors may view PHR depending on patient severity [7, 8]. To access the PHR, a secure modified ECC is presented. A one-time session key may encrypt and decode data. The session keys are sent to the user through e-mail. It is done via ECC.  Figure 2 depicts the proposed criticality analysis and secure cloud access architecture. PHRs are separated according to the urgency of patients who need rapid medical attention. The PHRs are separated into rings according to the patients' serious state [10, 11]. Protection ring 1 includes the patient records with the most critical condition. Doctors are alerted about critical patients.  Figure 2 represents the proposed architecture.

**Figure 3:**
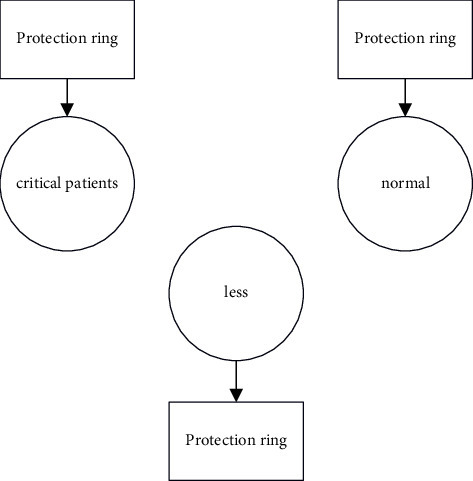
Protection ring formation.

Figure 3 depicts the protection ring formation of PHR. By using a ring-based approach, the separation of PHR is accomplished by labelling the criticality of patients as very critical, less critical, and normal.

Fuzzy inference system is used to analyze and identify the criticality of patients categorized as normal, less critical, and very critical. The increase in the number of linguistic variables in existing FIS results in the exponential growth of the fuzzy rule search space [12, 13]. This growth makes the learning process more difficult, and in most cases, it leads to problems of scalability in terms of the time and memory consumed and/or complexity with respect to the number of rules obtained and the number of variables included in each rule. The proposed optimized rule-based fuzzy inference system (ORFIS) overcomes the problem of scalability and complexity.

This database has 76 properties, but all reported experiments use just 14. To yet, only the Cleveland database has been utilized by ML researchers. The “goal” field indicates the patient has cardiac disease. It ranges from 0 (absence) to 4. The Cleveland database experiments focused on separating presence (1, 2, 3, and 4) from absence (value 0).

The patients' names and SSNs were recently deleted from the database and replaced with fictitious values.

The Cleveland database was “processed” in one file. This directory contains all four unprocessed files.

Only 14 attributes were used:

1. #3 (age)
2. #4 (sex)
3. #9 (cp)
4. #10 (trestbps)
5. #12 (chol)
6. #16 (fbs)
7. #19 (restecg)
8. #32 (thalach)
9. #38 (exang)
10. #40 (oldpeak)
11. #41 (slope)
12. #44 (ca)
13. #51 (thal)
14. #58 (num) (the predicted attribute).

### 3.1. Procedure of FIS

**Step 1 .**

Determine input and output variables

**Step 2 .**

Fuzzify the patient's heart disease attributes using input membership functions (triangular membership function).

**Step 3 .**

Determine a set of fuzzy rules

**Step 4 .**

Combine the fuzzified inputs (linguistic values) according to the fuzzy rules that is a collection of linguistic statements that describes how FIS should make a decision

**Step 5 .**

Combine all satisfied rules to get output distribution (max membership values)

**Step 6 .**

Defuzzify the output distribution to get a crisp result (centroid method)

### 3.2. Determining Input and Output Variables

FIS involves the process of determining the input and output variables to predict the criticality of heart disease [14, 15]. The attributes as per clinical data from the UCI Machine Learning Repository are taken as the set of input fields for heart disease prediction.

#### 3.2.1. Input Fields

Chest pain type, blood pressure, cholesterol, resting blood sugar, ECG, maximum heart rate, old peak (ST depression induced by exercise relative to rest), thallium scan, gender, and age are the essential attributes to predict heart disease criticality [16, 17]. The purpose of this data set is to diagnose the presence or absence of heart disease, given the results of various medical tests carried out on a patient, which used 10 input attributes and a single output attribute.

#### 3.2.2. Output Fields

The output field refers to the presence of heart disease in the patient. It is integer valued from 1 to 3 (distinguish presence (values 1, 2, and 3)): 1: very critical, 2: less critical, and 3: normal.

### 3.3. Calculation of Linguistic Values and Membership Function (Membership Value Assignment)

FIS includes the calculation of linguistic values for each input attribute, thereby determining the membership function and graph [18, 19]. The membership graph for the heart disease attribute is generated using the MATLAB fuzzy logic toolbox.

#### 3.3.1. Chest Pain (CP)

This input variable supports four chest pain types as shown in Table 2. Each chest pain type is a fuzzy set. In this field, fuzzy sets do not overlap, and sets are defined as crisp sets because the patient has only one type of chest pain at a time.

#### 3.3.2. Blood Pressure (BP)

This input variable is divided into four fuzzy sets, namely “low,” “medium,” “high,” and “very high” as shown in Table 3. The membership graph represents the blood pressure severity of the patients.


(1) $$\begin{array}{c} \mu_{\text{low}}=\left\{\begin{array}{cc} 1 & x<110\\ \frac{130-x}{20} & 110\leq x<130 \end{array}\right\},\\ \mu_{\text{medium}}=\left\{\begin{array}{cc} \frac{x-125}{11} & 125\leq x<136\\ 1 & x=136\\ \frac{153-x}{16} & 136\leq x<152 \end{array}\right\}, \end{array}$$

(2) $$\begin{array}{c} \mu_{\text{high}}=\left\{\begin{array}{cc} \frac{x-140}{15} & 140\leq x<155\\ 1 & x=155\\ \frac{170-x}{15} & 155\leq x<170 \end{array}\right\},\\ \begin{array}{c} \mu_{\text{veryhigh}}=\left\{\begin{array}{cc} \frac{1}{x-152} & x\geq 170\\ 18 & 152\leq x<170 \end{array}\right\}. \end{array} \end{array}$$

#### 3.3.3. Cholesterol (Ch)

**Figure 4:**
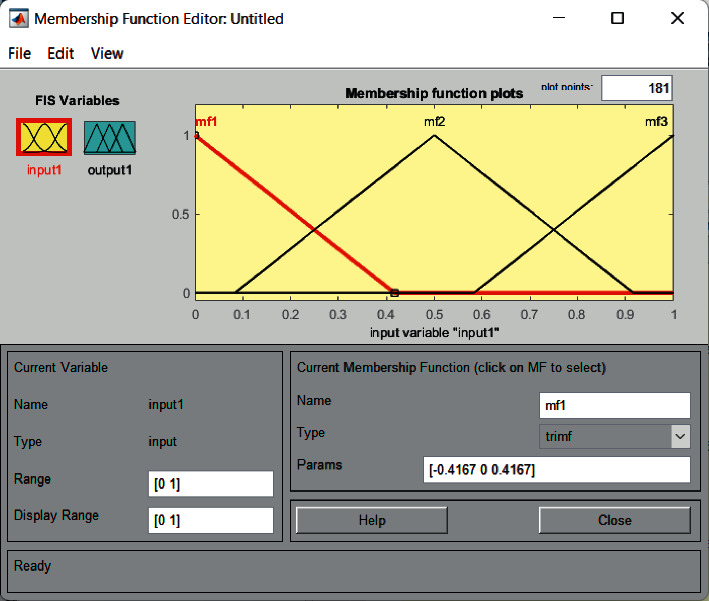
Membership graph for blood pressure.

Cholesterol has a salient effect on the result and can be changed easily. For this input field, the value of low-density lipoprotein (LDL) cholesterol is used. The cholesterol field has four fuzzy sets (low, medium, high, and very high) [9, 20]. The membership graph is shown in Figure 4. Membership expressions for cholesterol are defined in equations (1) and (2).

#### 3.3.4. Gender (G)

Value 0 represents a male patient, and value 1 represents a female patient for the attribute gender.

#### 3.3.5. Blood Sugar (BS)

**Figure 5:**
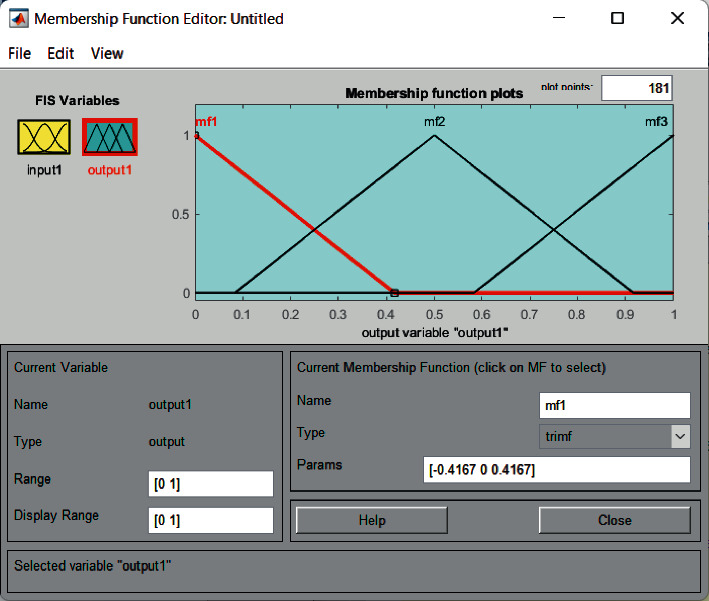
Membership graph for sugar.

Blood sugar is one of the most important factors in this system that changes the result. This input field has just one fuzzy set as shown in Table 4. A patient is diabetic if the value of blood sugar is higher than 120 (>120). The membership graph is shown in Figure 5. Membership expressions for cholesterol are defined in equations (3)–(5).


(3) $$\begin{array}{c} \mu_{\text{low}}=\left\{\frac{195-x}{50}145\leq x<195\right\},\\ \mu_{\text{medium}}=\left\{\begin{array}{cc} \frac{x-186}{24} & 186\leq x<210\\ 1 & x=210\\ \frac{245-x}{35} & 210\leq x<245 \end{array}\right\}, \end{array}$$

(4) $$\begin{array}{c} \mu_{\text{high}}=\left\{\begin{array}{cc} 1 & x=260\\ \frac{x-215}{45} & 215\leq x<260\\ \frac{306-x}{46} & 260\leq x<306 \end{array}\right\},\\ \begin{array}{c} \mu_{\text{veryhigh}}=\left\{\begin{array}{cc} \frac{1}{x-280} & x\geq 345\\ 65 & 280\leq x<345 \end{array}\right\} \end{array},\\ \mu_{\text{young}}=\left\{\begin{array}{cc} 1 & x<25\\ \frac{35-x}{10} & 25\leq x<35 \end{array}\right\},\\ \mu_{\text{mid}}=\left\{\begin{array}{cc} \frac{x-30}{5} & 30\leq x<35\\ 1 & x=35\\ \frac{45-x}{10} & 35\leq x<45 \end{array}\right\},\\ \mu_{\text{old}}=\left\{\begin{array}{cc} 1 & 42\leq x<50\\ \frac{x-42}{8} & x=50\\ \frac{58-x}{8} & 50\leq x<58 \end{array}\right\},\\ \mu_{\text{veryold}}=\left\{\begin{array}{cc} 1 & x\geq 61\\ \frac{x-55}{6} & 55\leq x<61 \end{array}\right\}.\; \end{array}$$

(5) $$\begin{array}{c} \mu_{\text{veryhigh}}=\left\{\begin{array}{cc} \frac{x-100}{20} & 100\leq x<120\\ 1 & x\geq 120 \end{array}\right\},\\ \mu_{\text{young}}=\left\{\begin{array}{cc} 1 & x<25\\ \frac{35-x}{10} & 25\leq x<35 \end{array}\right\},\\ \mu_{\text{mid}}=\left\{\begin{array}{cc} \frac{x-30}{5} & 30\leq x<35\\ 1 & x=35\\ \frac{45-x}{10} & 35\leq x<45 \end{array}\right\},\\ \mu_{\text{old}}=\left\{\begin{array}{cc} 1 & 42\leq x<50\\ \frac{x-42}{8} & x=50\\ \frac{58-x}{8} & 50\leq x<58 \end{array}\right\},\\ \mu_{\text{veryold}}=\left\{\begin{array}{cc} 1 & x\geq 61\\ \frac{x-55}{6} & 55\leq x<61 \end{array}\right\}.\; \end{array}$$

(6) $$\begin{array}{c} \mu_{\text{low}}=\left\{\begin{array}{cc} \frac{1}{x<95} & 110\leq x<153\\ \frac{140-x}{45}95\leq x<140 & \mu_{\text{medium}}=\left\{\begin{array}{cc} \frac{x-110}{43} & x=153\\ 1 & 153\leq x<195\\ \frac{195-x}{42} & 15\leq \end{array}\right\} \end{array}\right.,\\ \mu_{\text{young}}=\left\{\begin{array}{cc} 1 & x<25\\ \frac{35-x}{10} & 25\leq x<35 \end{array}\right\},\\ \mu_{\text{mid}}=\left\{\begin{array}{cc} \frac{x-30}{5} & 30\leq x<35\\ 1 & x=35\\ \frac{45-x}{10} & 35\leq x<45 \end{array}\right\},\\ \mu_{\text{old}}=\left\{\begin{array}{cc} 1 & 42\leq x<50\\ \frac{x-42}{8} & x=50\\ \frac{58-x}{8} & 50\leq x<58 \end{array}\right\},\\ \mu_{\text{veryold}}=\left\{\begin{array}{cc} 1 & x\geq 61\\ \frac{x-55}{6} & 55\leq x<61 \end{array}\right\}.\; \end{array}$$

(7) $$\begin{array}{c} \mu_{\text{high}}=\left\{\begin{array}{cc} \frac{x-155}{60} & 155\leq x<255\\ 1 & x\geq 215 \end{array}\right\},\\ \mu_{\text{young}}=\left\{\begin{array}{cc} 1 & x<25\\ \frac{35-x}{10} & 25\leq x<35 \end{array}\right\},\\ \mu_{\text{mid}}=\left\{\begin{array}{cc} \frac{x-30}{5} & 30\leq x<35\\ 1 & x=35\\ \frac{45-x}{10} & 35\leq x<45 \end{array}\right\},\\ \mu_{\text{old}}=\left\{\begin{array}{cc} 1 & 42\leq x<50\\ \frac{x-42}{8} & x=50\\ \frac{58-x}{8} & 50\leq x<58 \end{array}\right\},\\ \mu_{\text{veryold}}=\left\{\begin{array}{cc} 1 & x\geq 61\\ \frac{x-55}{6} & 55\leq x<61 \end{array}\right\}.\; \end{array}$$

#### 3.3.6. Fuzzy Rule Base

Fuzzy rules are linguistic IF-THEN constructs along with connectors “OR” or “AND” that have the general form “IF A THEN B,” where A and B are collections of propositions containing linguistic variables [1]. In effect, the use of linguistic variables and fuzzy IF-THEN rules exploits the tolerance for imprecision and uncertainty. In this respect, fuzzy logic mimics the crucial ability of the human mind to summarize data and focus on decision-relevant information [2]. The total number of rules as shown in equation (6) required to form a fuzzy rule base is defined as follows.  Table 5 represents the fuzzy rules.

(8) $$\begin{array}{c} N=\prod_{i=1}^{n}\left(\text{number of fuzzy values in fuzzy variable}\right). \end{array}$$

There are 82,944 fuzzy rules for heart disease diagnosis, and the rules are formed as listed in Table 5. Number of rules increases exponentially with the dimensions of the input space that results in the curse of dimensionality [3]. To overcome this existing issue in the fuzzy rule base, the optimized rule-based fuzzy inference system (ORFIS) is proposed and implemented in the PHR.

### 3.4. Optimized Rule-Based Fuzzy Inference System (ORFIS)

**Figure 6:**
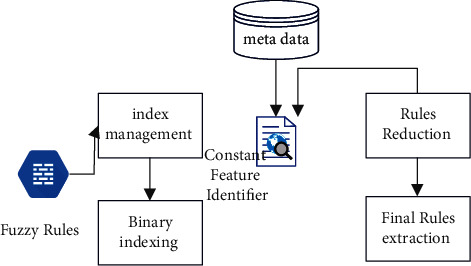
Fuzzy inference system.

Fuzzy rule base suffers from the exponential growth of the fuzzy rule search space that leads to scalability problems (in terms of the time and memory consumed) and/or complexity (with respect to the number of rules obtained and the number of variables included in each rule). As a result, recognizing rules that are satisfied from the rule base is still a work in progress. Selecting just those qualities from the data sets capable of minimizing rule search is done using the already constructed rule sets. With the number of reduced rules, satisfied rules are extracted from the rule base [4]. This algorithm overcomes the problem of rule search where the number of rules in the rule base is very high. The architecture diagram of the proposed ORFIS is shown. In the proposed ORFIS, fuzzy rules are sorted alphabetically that in turn generates the index for sorted fuzzy rules that contains constant attribute and the corresponding index reference [5]. The values of the constant attributes are acquired while adding the record and sent to the index, which returns the number of rules to be searched finally. The information about these constant attributes is present in the metadata repository. For example, each patient can have one type of chest pain at a time, which is denoted as 1 – typical angina, enough to search for the rules having only one type of chest pain [6]. This property is called rule reduction. This process will be repeated until all the attributes are forwarded to the diagnosis logic block. The proposed ORFIS for extracting rules from the rule base and the flowchart for ORFIS is shown in Figure 6.

The effectiveness of ORFIS is analyzed as follows: input I – set of fuzzy rules; 82,944 sample rules are formed; input II – search for rule; chest pain type: asymptomatic angina, blood pressure: low; cholesterol: medium; blood sugar: normal; ECG: hypertrophy; max heart rate: low; old peak: risk; thallium scan: normal, gender: female; and age: very old. In the proposed ORFIS, the algorithm produce index, which is used to generate the Index, has three constant attributes: the kind of chest pain experienced, the results of the thallium scan, and the gender of the person who is experiencing the chest pain. In the sample heart disease data set, there are a total of 82,944 rules that are connected with the chest pain type. The reference index generated using the B+ tree contains four indices, namely indexes 1–4 for each chest pain type, namely asymptomatic angina, atypical angina, nontypical angina, and typical angina that contains 20,736 rules each. Similarly, the next constant attribute in the rule set is thallium scan that includes three types: fixed defect, normal, and reversible defect. The generate index () algorithm takes index 1 with 20,736 rules as input resulting in 6,912 rules for each thallium scan type reference index 11. The third reference index for the constant attribute gender that has two values, namely male and female, contains 3,456 rules each. Thus, the rule reduction and extraction take place with the B+ tree index and constant attributes in the sample dataset. The optimized rule-based fuzzy inference system reduces the time of rule search and increases the performance. The above test case reduces the rule search from 82,944 rules to 34,56 rules, that is, the search time decreases in the ratio 24 : 1.

It is necessary to defuzzify the results of a fuzzy inference method before proceeding. Although fuzziness aids in the evaluation of rules, the ultimate output of a fuzzy system must be a discrete number to be considered valid. The fuzzy results that are produced cannot be utilized in the applications themselves. As a result, it is important to transform fuzzy values into crisp quantities in order to proceed with further processing. This single defuzzified value is formed as equation (9) using the aggregate output fuzzy set as the input for the defuzzification process, and the outcome is the same as the aggregate output fuzzy set. The technique of the centre of sums is much quicker than any other defuzzification approach. Instead of combining distinct output fuzzy sets, it uses an algebraic sum of those sets.

(9) $$\begin{array}{c} z^{*}=\frac{\int z\sum_{i=1}^{1}\mu_{c}\left(z\right)\text{d}z}{\int \sum_{z-1}^{1}\mu_{c}\left(z\right)\text{d}z}. \end{array}$$

The cipher points *C*_1_ and *C*_2_ are given by the following equations, respectively:

(10) $$\begin{array}{c} C_{1}=gS, \end{array}$$

where *g* is a random integer for each character and *S* is a random point on the elliptic curve.

(11) $$\begin{array}{c} C_{2}=D+\left(K_{2}+g\right)M_{1}-gM_{2}+M_{3}. \end{array}$$

The fundamental benefit of a B+ tree is that the leaf nodes are connected together; therefore, scanning the whole tree for all items needs just a single linear pass over all of the leaf nodes to complete the scan. In the case of a B+ tree, traversal of every level in the tree is required. This full-tree traversal, however, will most certainly result in more cache misses than the linear traverse of B+ leaves.

**Figure 7:**
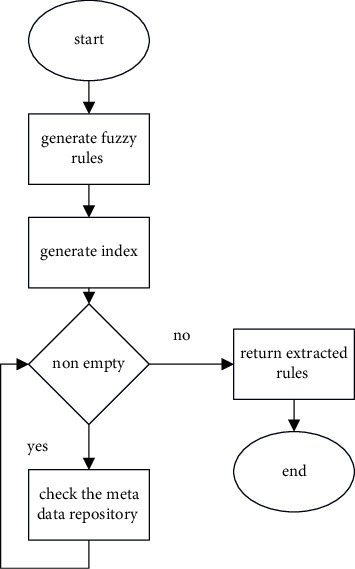
Flowchart for ORFIS.

The flowchart that has been presented is seen in Figure 7. The suggested PHR makes use of metadata to offer descriptions of the constant parameters of a data set that is based on ORFIS and is being used. Information regarding constant parameters such as chest discomfort, gender, and thallium scan may be found in the metadata repository for heart disease diagnosis. Because the values of these parameters are not fuzzy, the values of these parameters never overlap each other.

## 4. Results and Discussion

It is recommended that the proposed PHR be implemented and deployed in cloud bees, which are platforms that are standards-based, integrated, and scalable, allowing Java developers to create and deploy web applications in a cloud environment, as well as in on-premises environments. Cloud bees provide a platform as a service (PaaS) for developing, deploying, and managing web-based applications. It eliminates the requirement for the underlying information technology infrastructure to be kept up to date. We will discuss the experimental findings of the optimized rule-based fuzzy inference system for the heart disease diagnostic system and the performance of the RMBS combined with RBP in this section.

The suggested ORFIS's performance is assessed by altering the number of rules in the rule base in relation to the amount of time it takes to search for them. Experimenting with ORFIS is accomplished via the use of a large number of distinct rule bases. The performance of ORFIS is shown in Table 6. The time it takes for both the present FIS and the planned ORFIS to search for rules in the rule base is being tracked and analyzed. Upon examination of a large number of rule sets, it has been discovered that the suggested approach requires less time to search for rules in the rule base than the already available FIS.

FIS and ORFIS are both classified according to their membership grade, criticality, and search duration (see Table 6). The findings demonstrate that the suggested system is much quicker than the present FIS. Results of criticality analysis of a sample PHR are shown in the table below, with the patient condition being less critical (protection ring 2). When the proposed ORFIS is performed on the UCI sample data set, it takes 72 ms to find the crucial condition, while the current FIS took 1,721 ms to discover the critical condition.

**Figure 8:**
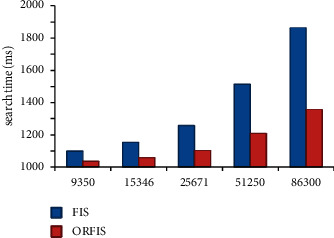
Performance of ORFIS.

Several existing fuzzy models, such as those developed by Mamdani, Sugeno, and Tsukamoto, have been implemented using the proposed ORFIS, and the runtime of these models has been assessed as shown in Figure 8. The findings demonstrate that the current models that include ORFIS perform much quicker than the existing models that do not include ORFIS.

**Figure 9:**
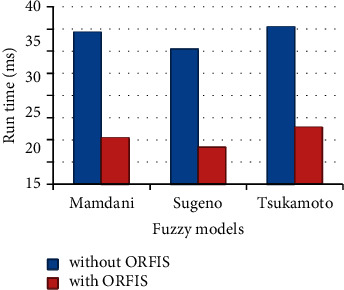
Performance of ORFIS with existing fuzzy models.

On the basis of multiple data sets from the UCI Machine Learning Repository, such as Breast-Cancer, UCI vital signs, and MHealth, the proposed ORFIS is compared with conventional models such as existing FIS and decision tree.  Figure 9 compares the accuracy of the proposed ORFIS with the accuracy of current classifiers for the different datasets under consideration. When compared to the current classifiers, it is discovered that the suggested ORFIS achieves an average accuracy rate of 96%.

**Figure 10:**
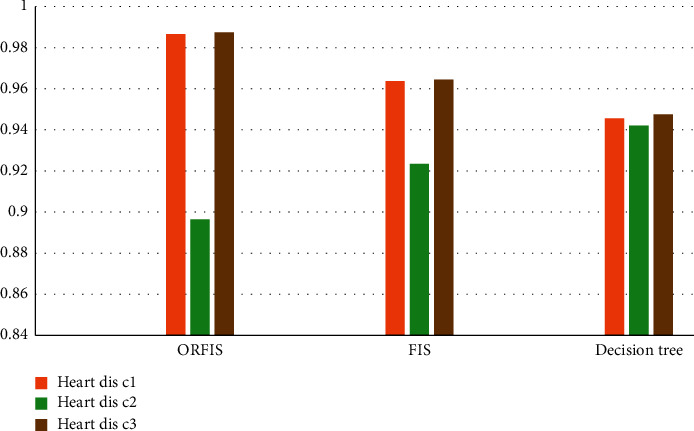
Comparison of ORFIS with existing classifier.

Figure 10 depicts the deployment of an application together with a criticality study performed using ORFIS. Following the establishment of their accounts, users will be given access to apps, databases, and repository locations. In order to get access to the proposed system, a URL for the application will be supplied. Sqlyog is a graphical user interface (GUI) application that is used to examine the databases existing in the cloud.

## 5. Conclusion

According to the findings of this study, security strategies for accessing personal health records are presented. It is addressed the current challenges in personal health records (PHR), such as privacy and security, access control, key management, and the time it takes to search for keywords as well as storage complexity. The suggested system incorporates criticality analysis with ORFIS, which minimizes the amount of time required to search for rules in a rule-based FIS. The suggested secure modified ECC algorithm is used to provide secure access to PHR. When a personal health record (PHR) is transferred to the cloud, security and privacy concerns arise. In order to address the security and privacy concerns, this study presented better attribute-based encryption algorithms for use in both the personal and public domains of personal health records (PHR). Implementing attribute and user revocation procedures in the relevant domains was advised to prevent unauthorized users from gaining access to data. Also being developed is enhanced tokenization (ET), which will be used to avoid the collusion attack in multiowner settings with a large number of users. It is recommended to use a graph-based access structure in conjunction with decentralized anonymous authentication of PHR in the cloud to avoid replay attacks and assure privacy preservation in a domain-based PHR environment. When compared to the present ABE, the suggested security techniques increase the efficiency of the PHR domain by 34.79% in terms of encryption, decryption, and key generation time. It has also been shown that the suggested scheme is noncollusive, and the security analysis assures that user privacy and confidence between users and authorities are maintained. This results in an increase in the security of PHR users in a multiowner environment. These security methods were tested in AVISPA, and the results demonstrated that the PHR configuration is immune to collusion attacks. Furthermore, the suggested ISABE increases the efficiency of the PHR system in the cloud by reducing the complexity of the storage system and the time required for PHR users to search for keywords in a multiowner cloud environment. This study Algorithm 1 and 2 therefore offers an efficient and secure PHR access in the cloud that can be installed in a real-time environment to assist the healthcare domain's operations.

## Figures and Tables

**Algorithm 1:**
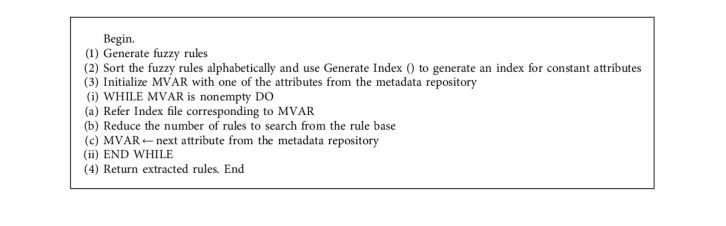
Fuzzy rules.

**Algorithm 2:**
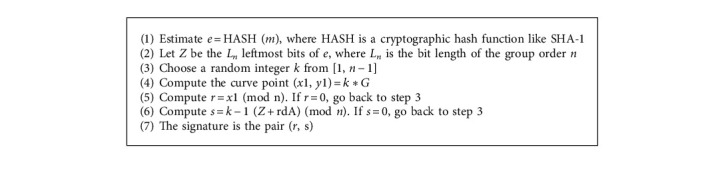
Pseudocode of attribute-based multisignature scheme.

**Table 1:** Existing methodology comparison.

Reference	Technique used	Research objective
[7]	Neuro-fuzzy inference system for diagnosis of malaria	Investigation of malaria using the neuro-fuzzy system for decision-making ability based on predefined rules and learning by the backpropagation algorithm

[8]	A fuzzy logic system with attribute ranking technique for risk-level classification of coronary artery heart disease (CAHD) in female diabetic patients	Fuzzy logic (Mamdani model) for risk classification of CAHD; it uses the attribute ranking technique (ART) for attribute selection

[10]	CDSS (clinical decision support system): risk assessment level weighted fuzzy rules are used to predict the development of heart disease	A weighted fuzzy rule-based CDSS is presented for the diagnosis of heart disease, by automatically obtaining knowledge from the patient's clinical data

[11]	A new approach for diagnosis of diabetes and prediction of cancer using adaptive neuro-fuzzy inference system (ANFIS)	ANFIS is used to improve classification accuracy and to achieve better efficiency; it examines the diagnosis of cancer and diabetes by training technique based on ANFIS for the early detection of sleep disorders

**Table 2:** Chest pain type.

Range	Value
1	Typical angina (typ)
2	Atypical angina (atyp)
3	Nontypical angina pain (NT)
4	Asymptomatic (asy)

**Table 3:** Fuzzy values of blood pressure.

Range	Linguistic values
<130	Low (L)
125–152	Medium (M)
140–170	High (H)
155>	Very high (VH)

**Table 4:** Fuzzy values of blood sugar.

Range	Linguistic values
<120	Normal (N)
>120	Very high (VH)

**Table 5:** Fuzzy rules and methods.

Fuzzy variables	No. of fuzzy values	Fuzzy variables	No. of fuzzy values
Chest pain type	4	ECG	3
Blood pressure	4	Old peak	3
Cholesterol	4	Thallium scan	3
Blood sugar	2	Gender	2
Maximum heart rate	3	Age	4

**Table 6:** Performance of ORFIS.

No. of rules	FIS rule search (rules)	ORFIS rule search (rules)	FIS search time (ms)	ORFIS time (ms)
9,350	9,350	389	197	7.96
15,346	15,346	639	306.92	12.96
25,671	25,671	1,070	513.42	21.58
51,250	51,250	2,135	1,025	42.88
86,300	86,300	3,595	1,726	72.08

## Data Availability

The data that support the findings of this study are available from the corresponding author on request.
